# Recent Developments in the Application of Nanomaterials in Agroecosystems

**DOI:** 10.3390/nano10122411

**Published:** 2020-12-02

**Authors:** Haleema Saleem, Syed Javaid Zaidi

**Affiliations:** Center for Advanced Materials (CAM), Qatar University, P.O. Box 2713, Doha, Qatar; haleema.saleem@qu.edu.qa

**Keywords:** nanomaterials, heavy metals, soil pollution, plants, environmental impact, toxicity

## Abstract

Nanotechnology implies the scientific research, development, and manufacture, along with processing, of materials and structures on a nano scale. Presently, the contamination of metalloids and metals in the soil has gained substantial attention. The consolidation of nanomaterials and plants in ecological management has received considerable research attention because certain nanomaterials could enhance plant seed germination and entire plant growth. Conversely, when the nanomaterial concentration is not properly controlled, toxicity will definitely develop. This paper discusses the role of nanomaterials as: (1) nano-pesticides (for improving the plant resistance against the biotic stress); and (2) nano-fertilizers (for promoting the plant growth by providing vital nutrients). This review analyzes the potential usages of nanomaterials in agroecosystem. In addition, the adverse effects of nanomaterials on soil organisms are discussed. We mostly examine the beneficial effects of nanomaterials such as nano-zerovalent iron, iron oxide, titanium dioxide, nano-hydroxyapatite, carbon nanotubes, and silver- and copper-based nanomaterials. Some nanomaterials can affect the growth, survival, and reproduction of soil organisms. A change from testing/using nanomaterials in plants for developing nanomaterials depending on agricultural requirements would be an important phase in the utilization of nanomaterials in sustainable agriculture. Conversely, the transport as well as ecological toxicity of nanomaterials should be seriously examined for guaranteeing its benign usage in agriculture.

## *Highlights* 

- Nanomaterials can be used as nano-pesticides.
- Nano-fertilizers are another promising application of nanomaterials.
- Nanomaterials can improve the plant growth.
- Proper risk assessment evaluations for nanomaterials are needed.

## 1. Introduction

Most scientists nowadays accept that nanomaterials (NMs) are one of the pillars of emerging science and technology in the twenty-first century. An increased number of NMs may reach the surroundings [1,2,3,4,5,6,7] by means of various paths, and, hence, the correlation between nanomaterials and environments has become a sensitive subject globally. Nanomaterials have at least one structural dimension at the nano-level, and these materials receive greater research attention due to their application probability in different zones of technology. Researchers nowadays can develop different materials at the nanoscale such as carbon nanotubes, nanofibers, nanoclays, and graphene with stronger, lighter, more noticeable chemical reactivity, and extended control on the light spectrum [8,9]. The increased understanding of the nanomaterial properties paves the way to develop advanced novel materials in the future and have the possibility to improve the quality of life. Nanomaterials are currently used in various fields, and several studies have been carried out on their environmental impact to the surroundings [10,11,12,13,14]. Nanomaterials are gradually becoming commercialized, starting to develop as commodities, and are utilized in several state-of-the-art technological applications as well as products, inclusive of an extensive variety of consumer products.

Engineered nanomaterials are those materials developed for a definite function or purpose and the intrinsic properties of these materials enable them to offer exceptional functions not attainable in another chemical state or form of the identical material; hence, their production and use have been increasing in an exponential manner. In the course of their life cycle, these materials can experience numerous transformations and reach a number of ecological compartments. They are anticipated to be seen in waters and sediments of fresh and marine water systems, soil, atmosphere, and biota [15,16,17,18,19].

In soil systems, nanomaterials can be used as nano-fertilizers as well as nano-pesticides [20,21,22,23,24,25]. Nano-fertilizers are nanomaterials that either act as additives/carriers (e.g., by compositing with minerals) for the nutrients or are nutrients themselves (macro- or micronutrients) [26,27,28,29,30]. These types of fertilizers can also be prepared by the encapsulation of nutrients within the nanomaterials [31,32,33]. Nano-fertilizers can improve the yield and quality of crops with increased nutrient usage efficiency, while lessening the production cost and thereby resulting in a sustainable agriculture [34,35,36,37,38]. The use of pesticide is a common practice in agriculture and several studies have been carried out for the advancement of effective pesticides [39,40,41,42]. Considering the environment sustainability, bio-based pesticides are being developed to reduce the dangerous impacts of artificial pesticides. However, the use of bio-based pesticides is restricted due to their low effectiveness against certain pests. In this scenario, nano-pesticides have proved to be promising in overcoming the limitations of synthetic as well as bio-based pesticides [43,44]. Very slow degradation together with controlled discharge of dynamic components by nano-pesticides contribute to efficient pest control over a prolonged time [45,46,47]. Nano-pesticides have the ability to conserve energy and water because they are used in lower quantities and less frequently as compared to traditional pesticides [48,49,50,51].

Despite all the benefits of the use of nanomaterials in the soil system, nanomaterials can also lead to some negative impact on the plants and soil organisms [52,53,54]. This review paper focuses on the positive and negative impacts of nanomaterials in the soil ecosystem. In this paper, we examine the advantages and disadvantages of nano-fertilizers and nano-pesticides such as nano-zerovalent iron, iron oxide nanoparticles, titanium dioxide (TiO_2_), nano-hydroxyapatite, carbon nanotubes (CNTs), and silver- and copper-based nanomaterials in the soil environment. The ability of nanomaterials to stimulate the growth of plants by helpful effects on biomass or increase root yield, shoot growth, and seed germination are also examined. In the end, the current regulations on the nanomaterial toxicity levels in different countries are also briefly reviewed. Clearly, vast research activities have been dedicated to further developments in the application of nanomaterials in different environmental remediation applications. Thus far, studies discussing the most recent progresses in the application of nanotechnology in soil system, particularly as nano-pesticides and nano-fertilizers, have been very limited. Correspondingly, we systematically analyze the hazardous impacts of the nanomaterials on soil organisms and humans. The data presented in this study will permit scientists outside and within the nano-biotechnology field to properly choose nanomaterials as preparatory materials for agricultural use.

## 2. Nanomaterials and Its Different Types

**Figure 1:**
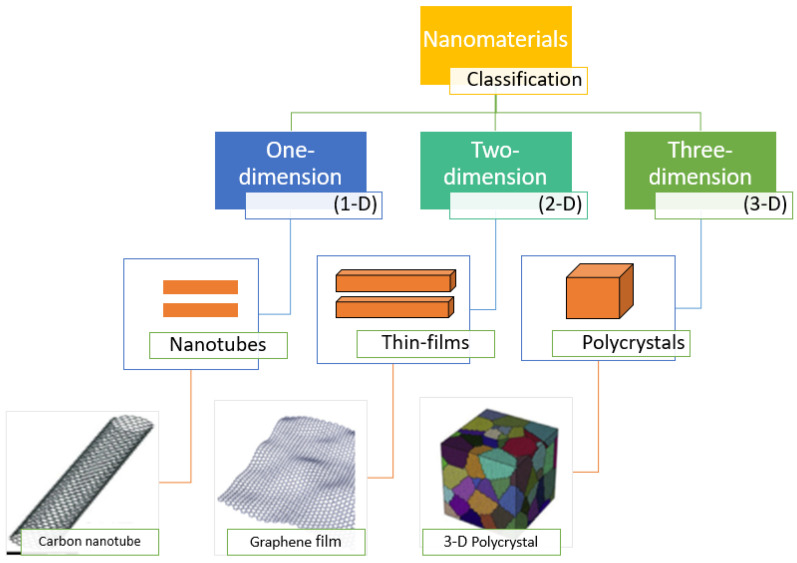
Diagrammatic portrayal of 1D, 2D, and 3D nanomaterials.

Nanomaterials are structures with dimensions of 1.0–100.0 nm [55]. Nanomaterials are different from their bulk equivalents because of their high surface-area-to-volume ratio and possible presence of quantum effects [56]. Numerous researchers are trying to develop improved nanomaterials with advanced properties and applications. According to their construction, the nanomaterials are presently classified as: (a) carbon-based; (b) composites; (c) dendrimers; and (d) metal-based [57]. Nanomaterials can also be categorized on the basis of their dimensionality: (1) one-dimensional (1D) nanomaterials; (2) two-dimensional (2D) nanomaterials; and (3) three-dimensional (3D) nanomaterials (Figure 1) [58]. One-dimensional nanomaterials are rod- or needle-like materials with length in the range from 100 nm to 10 μm and include nanowires, nanorods, and nanotubes. Two-dimensional nanomaterials show plate-like shapes inclusive of nanolayers, nanofilms, and nanocoatings. 0D, 1D, and 2D nanomaterials can be utilized on a substrate or they can be distributed in solid or fluid matrices. 3D nanomaterials have three arbitrary dimensions and a multilayer nano-crystalline framework, which might consist of multi-nanolayers, nanowire bundles, bulk powders, dispersions of nanoparticles, and nanotubes.

In soils, the naturally present nanoparticles are iron oxides, organic matter, clays, etc., and they perform a significant role in bio-geochemical processes. For several years, soil colloids have been researched with regard to their impact on soil development and soil structural behavior. Of special importance to synthesized nanomaterials, soil colloids as well as other porous media might assist the mobility of pollutants in soils, among other media. These pollutants could be transferred as soon as the conditions for colloidal transportation are satisfied. As an illustration, natural soil colloids are noted to be vectors for metal transportation through soil profiles.

## 3. Ecological Interactions of Nanomaterials

The biggest challenge of nanotechnology is the advancement of effective, state-of-the-art nanomaterials that consolidate approaches for responsible and sustainable production and guarantee their satisfactory and safe utilization and clearance. With the increased use of nanomaterials, there is a growing concern about the unintentional adverse impacts of the nanomaterials to the surroundings. Understanding the toxicological profiles of nanomaterials and their interactions at the interface of nano-bio-ecosystems is required for projecting and predicting their actual impacts on the sustainability of the planet [59].

The terrestrial environment is regarded as an ecosystem that, through indirect and direct pathways, is polluted and/or exposed to nanowaste from manufacturing production and consumption. Generally, nanomaterials have the ability to interact with the surroundings, abiotic and biotic, soon after their introduction into the surroundings. The aforementioned nano-bio-ecosystem interface typically includes the collaboration and transformation process of the nanomaterials, which are affected by their intrinsic features and the environment dynamics [60].

Soil consists of liquids, solids (organic matter and minerals), and gases, and soil is the main portion of the terrestrial surface. It is regarded as a complicated and extremely dynamic system (having particulate material and natural colloids) which contributes pronounced biotic diversity. The interaction between soil matrix and nanomaterials can happen in both aqueous and solid fractions. The pores present in soils (micropores and macropores), which openly control air diffusion and water relations, could operate as sites for sorption in addition to the biotransformation of nanomaterial. Furthermore, biopores developed by earthworms, other organisms, and roots, which are perfect habitats for microbes, also affect the transportation and discharge of nanoparticles [61].

Soil mineral structures demonstrate distortions and substitutions of atoms inside the crystal structure that lead to the electric unbalance of the minerals. Kinetic analysis indicates that heteroaggregation could happen in soils because of the interactions of positively charged sites of soil components and negatively charged nanomaterials. Consequently, various categories of soils with distinct organic matter loadings and clay fraction are also a significant factor which might influence the interaction between nanomaterials and microbial communities [62]. An additional significant factor which happens at the nano-bio-ecosystem interface is the nanomaterial dissolution, which is contingent upon its surface area, charge, surface chemistry, aggregation state, morphology, size, and composition. The dissolution affects its motion in the surroundings. Furthermore, environmental conditions normally affect the nanomaterial fate by changing their physicochemical properties or being transformed by sorption, biological processes, or chemical processes.

The various processes that manage the condition of nanomaterials in soil are the same as in aqueous systems. In the case of certain nanomaterials, the dissolution process might be extremely significant, since it degrades them such that the fate and bioavailability are aligned. It was illustrated that bulk zinc oxide dissolves very quickly in soils [63,64], thus zinc oxide nanomaterials are expected to be ephemeral in soil except when coated with any agent for dissolution restriction. In [65], the authors studied the fate of nanomaterials in soil and carried out a study comparing the characteristic pH and ionic strength of soil saturated extract with respect to characteristic critical coagulation concentration of nanomaterials. They concluded that homoaggregation of nanomaterials will be slow in most soils because the ionic strength and pH of most soil solutions are under the acute coagulation level of most nanomaterials. Heteroaggregation is considered to be extremely significant in soils, as the soil porewaters generally consist of greater contents of natural colloids in suspension. The authors of [64] revealed the deficiency of nanomaterial-specific impacts in soils, with bulk zinc oxide and nano zinc oxide functioning in the same way, with respect to dissolution, fate, and dissolution. The nano zinc oxide could dissolve and be transformed into a blend of species such as Zn-substituted ferrihydrate, Zn-cysteine, Zinc sulfide, zinc phosphate, and Zn^2+^ adsorbed to mineral surfaces.

In the past decade, studies of nanomaterial transport by means of soils have advanced from the use of inert stationary phases in columns, thus improving the understanding of the possible transportation of nanomaterials in soil systems [66]. Carbon nanotubes have been demonstrated to be reserved in soil because of their higher aspect ratio, resulting in substantial strain [67]. Further, the nanomaterial fullerene can also be reserved in soil, possibly by stronger interfaces with the organics present in soil [68].

## 4. Nanomaterials (NMs) in Soil Environment and Impact on the Plants

Engineered nanomaterials could come into soils by means of various sources and paths, e.g., the utilization of sewage water, plant protection products, fertilizers, biosolids, and floodplains. Soil is a multilayer matrix, and it is a multifarious interface between organisms and various matters. Nanomaterials might enter through the soil pores and get attached to the soil particles because of their greater surface area. In the following sections, we examine the positive and negative impacts of some nanomaterials in the soil environment.

### 4.1. Positive Influence of NMs in the Soil Environment and on the Plants

Table 1 shows some of the positive impacts of nanomaterials on plants. In the following section, we examine the role of nano-sized materials as: (1) nano-pesticides (for improving the plant resistance against the biotic stress); and (2) nano-fertilizers (for promoting the plant growth by providing vital nutrients). All aforementioned categories of nanomaterials are potential representatives of advanced agrochemicals that are capable of meeting the simultaneous challenges of environmental protection and food obtainability. Researchers needed to consolidate the benefits of nanotechnology and phytoremediation as an advanced potential pollutant restoration method technique [69].

#### 4.1.1. Nanomaterials as Nano-Fertilizers

Micronutrients have a significant role in the protection of crop plants against diseases [75]. Moreover, by destroying pathogens and enhancing stress tolerance, an additional approach for suppressing the crop disease is by improving the nutritive standing of plants with nanomaterials. The availability of minerals in the soil could impact the plant development by controlling the availability of water and heavy metal accumulation in the soil that could lead to extreme toxicity. The authors of [76] prepared foliar micronutrients sprays consisting of metallic oxide nanomaterials such as ZnO, nickel(II) oxide, MnO, ferrous oxide, copper(II) oxide, and aluminum oxide and tested on pathogen-diseased tomatoes and eggplants in a field experiment. They observed that nanomaterials consisting of copper(II) oxide, manganese oxide, and zinc oxide improved the yields of eggplant and tomato, despite the infection of the plants with *Fusarium wilt* fungus, by decreasing the range of disease. Therefore, these nanomaterials could function as both nano-fertilizers and nano-pesticides. Several studies have confirmed that nano-fertilizers can improve the biomass production [77,78].

Abiotic stress, excess salinity, heat, drought, cold, oxidative stress, and nutrient deficits are the main reasons for crop loss globally and lessen the average productivity of main crop plants by more than 50%. The abiotic stress results in molecular, biochemical, physiological, and morphological variations in plants, which can severely influence their productivity and growth. In recent times, numerous studies have confirmed the beneficial influences of nanomaterials on lessening the heavy metal stress in plants. In [79], NHAP was used as nano-fertilizer in soybean, and the results show almost 6.5 times increased ground biomass. In [77], it was found that the nano-fertilizers stimulated the plants to adsorb increased quantities of nutrients and develop double the root biomass, as compared to the control.

We analyze the use of different nanomaterials such as iron oxide, nano-zerovalent iron, titanium dioxide, cerium oxide, nano-hydroxyapatite, and copper nanoparticles as nano-fertilizers in agroecosystems in the following section.

##### Iron Oxide Nanoparticles as Nano-Fertilizers

Maghemite (γ-Fe_2_O_3_) and magnetite (Fe_3_O_4_) are two common types of iron oxide nanoparticles. Various forms of iron oxide demonstrate dissimilar properties. It is essential to examine the long-term influence of magnetite and maghemite nanoparticles on the plants grown in soil.

The authors of [80] analyzed the influence of citrate-coated nanoparticles (NPs) on the germination and early development of *Quercus macdougallii* (oak). They illustrated that the citrate-coated magnetite nanoparticles exhibited catalytic activity similar to peroxidase. The utilization of the nanoparticles enhanced the rate of germination by 33%, as compared to the control. Moreover, the citrate-coated magnetite nanoparticle treatments improved the chlorophyll concentration, growth, and dry biomass concentration. The information gained from this work suggests that these nanoparticle treatments could be utilized to increase conservation and reforestation of endangered forestry species. Recent studies have confirmed that the impacts of NMs on plants are species-specific and adaptable among plant species [81].

The authors of [82] demonstrated that magnetite and maghemite nanoparticles contributed positive effects on the content of Vitamin C content. In addition, they noted that both magnetite and maghemite nanoparticles started to stimulate the development of plants and increase the content of chlorophyll at a certain stage of exposure. As reported in [83], Fe_3_O_4_ nanoparticles could be used as an efficient source of iron for *Arachis hypogaea* plants, substituting the traditional iron sources. The iron(II, III) oxide nanoparticles treatment enhanced the root length, plant height, and Fe and chlorophyll contents of the plants. It also controlled the activity of antioxidant enzymes and phytohormone levels (reduction in abscisic acid and enhancement in gibberellic acid content).

##### Nano Zerovalent Iron as Nano-Fertilizers

**Figure 2:**
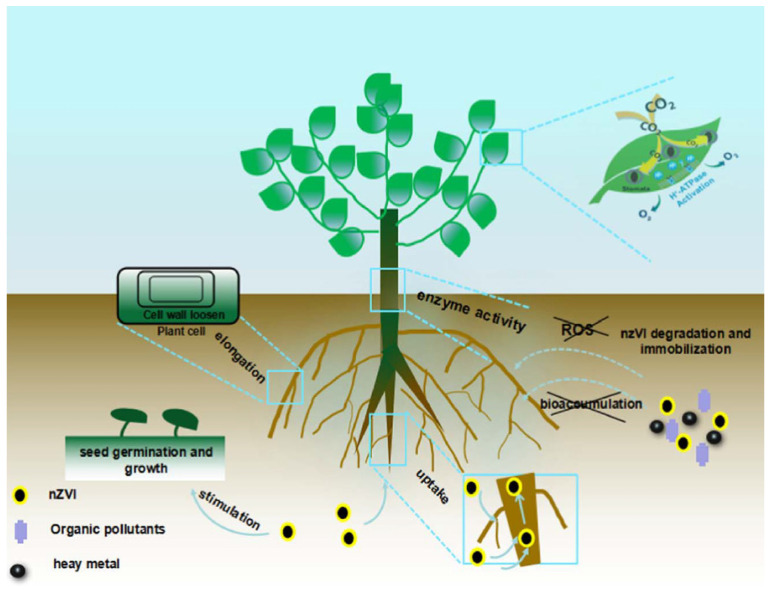
Conceivable impact of nZVI particles on plants. Reproduced with permission from [87], Elsevier, 2018.

An extremely low concentration of nano zerovalent iron (40–80 µmol/L) could stimulate the rate of germination of peanut seeds. Consequently, the shoot and root length of the peanut seedlings were further enhanced with additional exposure to the nano zerovalent iron [74]. In [84], it was noted that the exposure of plants to nano zerovalent iron concentration of 500 mg/L could increase the elongation of root because this nanomaterial promotes the cell wall loosening. In [70], lower concentrations of nano zerovalent iron particles were assessed for their growth enrichment possibility as a seed priming agent in *Oryza sativa cv. Gobindabhog*, an aromatic rice cultivar. Seeds were primed with various concentrations of nZVI (160, 80, 40, 20, and 10 mg L^−1^) and permitted to grow for about 14 days. It was noted that priming with lower doses of nano zerovalent iron enhanced seedling vigor, as indicated by enlarged shoot and root length and increased biomass and photosynthetic pigment content. The conceivable impact of nZVI particles on plants is demonstrated in Figure 2. The results confirm that nZVI at low concentrations could be utilized with hardly any adverse impacts on plants and consequently be appropriate for consolidation with plant remediation. nZVI with biochar could efficiently decrease the upward translocation of cadmium in plants, which is beneficial to the development of plant [85]. Silver can increase the biological functions of salt marsh plants and is beneficial for plant rehabilitation [86].

##### Titanium Dioxide Nanoparticles as Nano-Fertilizers

**Figure 3:**
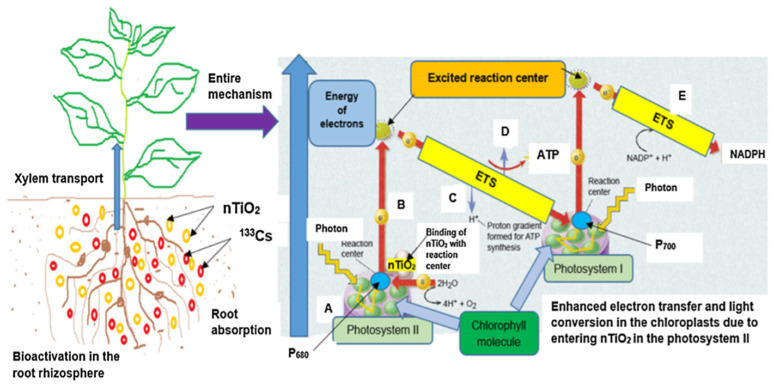
Potential mechanism for improving the accumulation of ^133^Cs by the addition of nano-titanium dioxide. Reproduced with permission from [71], Elsevier, 2018.

In addition to the nanomaterials stated above, titanium dioxide nanoparticles are extensively utilized. The authors of [71] examined the impacts of titanium dioxide NPs on the uptake of cesium by soybean plant, which was carried out in a plant growth chamber with the addition of various concentrations of ^133^Cs to the soil. They observed that, with the utilization of titanium dioxide nanoparticles, the buildup of ^133^Cs recognized in the shoot was greater as compared to the root. The potential mechanism for improving the accumulation of ^133^Cs by the addition of nano-titanium dioxide is demonstrated in Figure 3. Nano-titanium dioxide particles of smaller size (<5 nm) can develop a covalent bonding with the majority of the non-conjugated NOM and be translocated into the tissue and cells of plants. This study confirmed that the use of TiO_2_ nanoparticles in cesium polluted soil can considerably improve ^133^Cs uptake and its accumulation in plants. In addition, it was also found that nano-titanium dioxide can enhance plant protein content.

Cadmium is considered the most harmful trace metal in the surroundings because of its increased solubility in water. The authors of [88] demonstrated that, with a rise in the nano-titanium dioxide concentration in the soil, the cadmium buildup in roots and buds of soybean (Gycin max (L.) Merrill) plants enhances, and increased buildup happens in the root relative to the bud. The aforementioned work confirmed that the utilization of nano-titanium dioxide particles enhances the bioaccumulation of cadmium from polluted soil. All soybean seeds analyzed in this study germinated more quickly subsequent to the nano-titanium dioxide treatments relative to control. The test results of this work confirm that the utilization of nano-titanium dioxide particles can reduce cadmium stress and enhance the uptake of cadmium in soybean plants. Consequently, this method can be suggested for industrial-scale utilization and in real field situations. Other research works have confirmed that nano titanium dioxide stimulated an increment in the average root lengths of plants such as onion and cucumber [89]. As a result, nano-titanium dioxide can develop the capability for plants to absorb the contaminants. Abdel et al. [90] noted that the incorporation of 0.01% nano-titanium dioxide to the soil could considerably enhance the area of the leaf, dry weight of root, and stem length of leguminous crops.

##### Cerium Oxide and Zinc Oxide Nanoparticles as Nano-Fertilizers

Cerium oxide nanoparticles are commonly used in mechanical planarization, catalytic applications, and fuel additives [91]. Rico et al. [73] carried out a soil microcosm research work to inspect the influence of cerium oxide NPs on the productivity, physiology, and macromolecular composition of barley. The test results confirm that cerium oxide nanoparticles (500 mg kg^−1^) stimulated the development of plants, bringing about 331% enhancement in shoot biomass, relative to the control. Cerium oxide nanoparticles significantly enhanced the chlorophyll and biomass content, and the Ce accumulation was also accompanied by improved storage of nutrients (Cu, Zn, Fe, S, Mg, Ca, K, and P) in grains. The treatment using these nanoparticles did not aggravate the leaf oxidative stress; however, the productivity was compromised at the maximum cerium oxide nanoparticle treatment (500 mg kg^−1^). Wang et al. [92] examined the mutual effects of two frequently used engineered nanoparticles, zinc oxide nanoparticles and cerium oxide nanoparticles, and two inorganic species of arsenic on their uptake and buildup in rice seedlings in a hydroponic system. They noted that neither the entire arsenic nor the specific species of arsenic in rice tissues was remarkably varied by cerium oxide nanoparticles. The authors of [78] evaluated the effectiveness of the use of nanoparticles in *Phaseolus vulgaris* L. cv. considering the total biomass and the nitrogen assimilation. The test results confirm that the best concentration for promoting a rise in biomass was 25 mg/L zinc oxide nano-fertilizer.

##### Nano-Hydroxyapatite (NHAP) as Nano-Fertilizers

Lead (Pb) is considered to be one of the abundant soil pollutants. Nano-hydroxyapatite (NHAP) can adsorb lead and decrease its mobility in soils. The approach to enhance lead phytoremediation is extremely serious in view of the trouble in phytoextraction of lead because of its low solubility and increased retention on soil particles. The significant prospects of nano-fertilizers should be confirmed experimentally in different field environments. The authors of [93,94,95,96] confirmed the potential use of NHAP as nano-fertilizers. In the aforementioned work, the influence of various NHAP solutions stabilized using the carboxymethylcellulose were examined on growth of seedling, germination, and *Solanum lycopersicum* L. metabolism. The study confirmed that the percent germination of *S. lycopersicumis* was not affected by enhancing the NHAP concentrations, whereas root elongation was stimulated very strongly. Moreover, the team noted that the tomato plants grown in hydroponics with NHAP did not suffer from any phytotoxic effects.

Several other studies also confirmed the potential use of NHAP-based nanoparticles for promoting plant growth [97,98]. Yang et al. [99] determined the impact of NHAP of various sizes on the availability of cadmium for *Apium graveolens* L. They noted that the use of NHAP had the greatest effect on the immobilization of cadmium. and the cadmium absorbed by *Apium graveolens* L. reduced by enhancing the application rate of NHAP. The influence of the utilization of this nanomaterial on lead phytoextraction by ryegrass (*Lolium Perenne* L.) was examined in [72]. The test results demonstrate that the use of 5 g/kg nano-hydroxyapatite on lead-polluted soils remarkably enhanced the biomass of ryegrass. The lead removal rates from the contaminated soil by *ryegrass* were increased noticeably subsequent to the addition of NHAP. The test results confirm that nano-hydroxyapatite was appropriate for use in in-situ lead-contaminated soils for remediation. The results from the aforementioned research provided beneficial information about the influence of nano-hydroxyapatite on the removal of lead, which plays a significant role in phytoremediation. The authors of [100] illustrated that nano-hydroxyapatite can effectively decrease the mobility and bioavailability of lead. The incorporation of nano-hydroxyapatite significantly decreased the reducible and exchangeable segments of lead in soil and converted these segments into an oxidizable and residual lead, thereby restricting its mobility as well as its bio-accumulating ability. Nano-hydroxyapatite can have a significant role in controlling and mitigating the risks of lead contamination in the environment and organisms. The lead concentrations of roots and shoots reduced, and soil pH was not changed remarkably with the inclusion of nano-hydroxyapatite; furthermore, this nanomaterial stimulated the development of ryegrass and tartaric acid secretion. The aforementioned also confirmed that the use of nano-hydroxyapatite was advantageous to plant growth and did not show any adverse influence on the surroundings. Thus, the test results in the aforementioned study confirm that nano-hydroxyapatite can immobilize lead in polluted soil efficiently and can benefit ryegrass growth. Plants can accumulate lead and distribute it in the plant parts which are harvestable. A phytoremediation field test utilizing nanomaterials and ryegrass was carried out in [101]. The test results demonstrate that ryegrass has potential as a plant for the phytoremediation of lead-polluted soil. This plant species has comparatively high biomass and is cosmopolitan and ubiquitous. The lead phytoextraction possibility by ryegrass remarkably enhanced with the application of NHAP, relative to control. The combined effects of a 0.2% nano-hydroxyapatite soil utilization with ryegrass could be efficient for treating higher concentrations of lead in the soil. Repeat harvesting and testing of the ryegrass indicated that the phytoremediation by ryegrass of lead-contaminated soil is efficient. NMs coupled with ryegrass promoted the phytoextraction, and, hence, this technique could be a green substitute to traditional costly and ecologically unfriendly physical-chemical methods.

##### Copper Nanoparticles as Nano-Fertilizers

**Figure 4:**
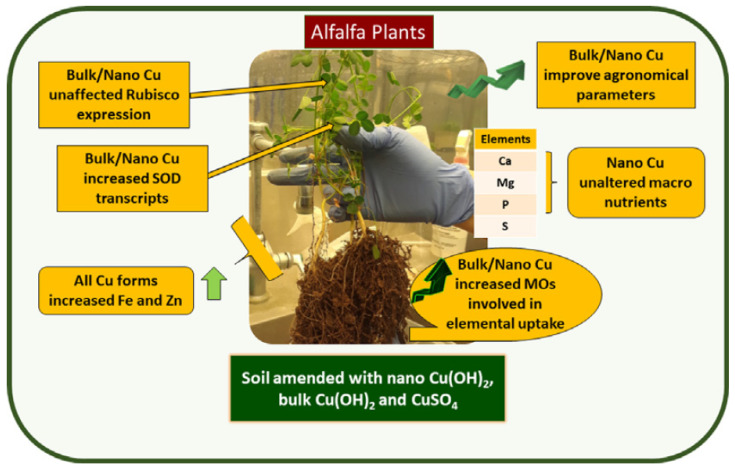
Alfalfa (Medicago sativa) in potting mix amended with bulk, nano, and ionic copper compounds at 80 and 280 mg Cu/kg. Reproduced with permission from [36]. Elsevier, 2020.

The latest application of copper-based nanoparticles in agriculture has confirmed efficient physiological performance, improving the yield of crops. These nanoparticles can effectively replace the conventional copper bulk materials, thereby reducing the copper content in the environment. ISeveral studies have been conducted recently on this topic, confirming that commercial copper oxide nanoparticles and custom-prepared copper nanosheets can substantially reduce fungal infections and increase the productivity of foliar-treated watermelon plants at 100.0–250.0 and 10.0–25.0 ppm, respectively [102]. The authors of [36] grew alfalfa in potting mix using bulk copper, nano copper, and ionic copper compounds and then examined the performance of plant at physiological and molecular levels. They noted that plants that underwent treatment with bulk and nano copper showed improved agronomical response. The phosphorus and sulfur contents were decreased in ionic and bulk copper exposed plants, relative to controls. The overall results (Figure 4) indicate that copper nanoparticles can improve the physiology of alfalfa and has promise as nano-fertilizer. Several other studies also confirmed the potential use of copper-based nanoparticles for promoting the plant growth [103,104].

#### 4.1.2. Nanomaterials as Nano-Pesticides

**Figure 5:**
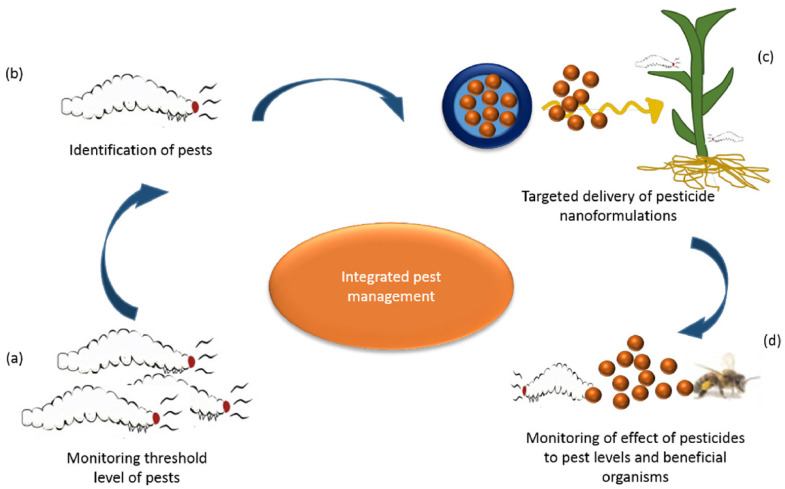
Possible application of nanotechnology in the management of pests: (**a**) monitoring threshold pest levels; (**b**) pest identification; (**c**) pesticide delivery; and (**d**) monitoring the effects of nano-pesticides on the level of pests. Reproduced with permission from [112]. Elsevier, 2018.

Biotic stress, such as pathogen infection, is a significant factor influencing the production of crops. Even though the utilization of pesticide has enhanced agricultural productivity and production, conventional pesticides pose environmental and health risks. The use of nanotechnology in the form of nano-pesticides in agriculture is still in its early development stage. Scientists define nano-pesticides as pesticide formulations or products consisting of engineered nano-sized materials as active ingredient and possessing biocidal features. Numerous nanomaterials such as silver nanoparticles show excellent pest-control and antibacterial functions [105,106,107,108,109,110,111]. Nano-pesticides behave differently from traditional pesticides. These pesticides have high efficiency and offer increased crop productivity by greater yields and reduced input expenses by lessening the labor cost and waste. Figure 5 presents the promising utilization of nanomaterials for managing pests [112]. Table 2 presents different studies based on nano-pesticide application. In the following section, we examine the use of different nanomaterials such as silver nanoparticles, carbon nanomaterials, and copper-based nanomaterials as nano-pesticides in agroecosystems.

##### Silver Nanoparticles as Nano-Pesticides

The increasing numbers of pests and fungi resistant to the prevailing chemical pesticides have emphasized the need for developing advanced methods for the protection of crops. Silver nanoparticles, with their widespread range of antibacterial activities, have gained substantial attention as a prospective nano-pesticide used in agriculture. Numerous in vitro studies confirmed the activity of silver nanomaterials against the development of various pathogens [116]. The silver nanomaterial toxicity mechanism has not been entirely resolved yet; conversely, it can be noted that it is mainly developed from the silver ion discharge. In [117], silver nanoparticles developed on a double-stranded DNA and graphene oxide were demonstrated to stop the activities of *Xanthomonas perforans* both in planta and in vitro. Ocsoy et al. [117] manufactured DNA-directed silver nanoparticles developed on graphene oxide (GO) and established that these composites at 16 ppm remarkably reduced the activity of cultured pathogen *Xanthomonas perforans*. The bacterium leads to tomato bacterial spot, resulting in 10–50% decrease in productivity. Comparable results were attained in a greenhouse test using the aforementioned type of composites at 100 ppm. Furthermore, silver nanoparticles have presented activity against the commonly seen soil borne organism nematodes. Cromwell et al. [118] observed that, when *Meloidogyne* spp. was subjected to silver nanoparticles (30.0–150.0 mg/mL) for six days, 99.0% of the nematodes died. In a field analysis, silver nanoparticles at 150 mg/mL concentration decreased the nematode number by 92% on Day 4 and 82% on Day 2.

Relative to chemical pesticides, the green-synthesized silver nanoparticle can be prepared in an ecologically friendly way. Plant extract or bacterial extract consisting of numerous metabolites functions as reducing agent and capping agent at the time of preparation of the silver nanoparticles. Mishra et al. [119] employed a plant-growth promoting rhizobacterium *Serratia* sp. for biosynthesizing silver nanoparticles. Under the greenhouse conditions, these biosynthesized silver nanoparticles showed stronger antifungal activity against the spot blotch pathogen of wheat, *Bipolaris sorokiniana*.

Considering the studies examining the application of silver-based nanoparticles as nano-pesticides, it could be confirmed that these engineered nanomaterials are effective against different pathogens in plants. On the other hand, most works did not compare their results with traditional pesticides, and most were carried out in controlled environments. Therefore, it is necessary to perform field trials to confirm the optimum conditions for effective functioning of these nano-pesticides.

##### Carbon-Based Nanomaterials as Nano-Pesticides

Wang et al. [115] inspected the antifungal efficiency of six carbon-based nanomaterials (at 500, 250, 125, and 62.5 ppm), namely single-walled carbon nanotubes, multi-walled carbon nanotubes, fullerene (C60), activated carbon (AC), reduced graphene oxide (rGO), and graphene oxide (GO), against two significant plant pathogen fungi (*Fusarium poae* and *Fusarium graminearum*). The nanomaterials were coincubated using *Fusarium poae* and *Fusarium graminearum* for 12 and 5 h without any light. Resilient antifungal activity was shown by single-walled carbon nanotubes (500 ppm), followed by multi-walled carbon nanotubes (500 ppm), GO (500 ppm), and rGO (500 ppm), while the activated carbon at the tested concentration range displayed no antifungal influence. At 500 ppm, fullerene showed spore germination of *Fusarium graminearum* but not with *Fusarium poae*. They confirmed that the fundamental mechanism of the antifungal activities of the carbon nanomaterials include water uptake inhibition and plasmolysis induction.

##### Copper Nanomaterials as Nano-Pesticides

The antifungal and antibacterial activities of copper ions are renowned, and copper hydroxide nanoparticles are the active component in Kocide 3000, a commercially available pesticide. Antimicrobial activities of copper nanoparticles have been demonstrated against the plant fungal pathogens *C. lunata*, *A. alternata*, *F. oxysporum*, and *Pdestructiva* [120] and the bacterial pathogens *Scherichia coli* and *Bacillus subtilis* [121]. A study comparing the antibacterial efficiency of copper nanoparticles versus the traditional fungicide bavistin confirmed the superior efficiency of copper nanoparticles. In addition, Borgatta et al. [102] recently compared the efficiency of copper(II) oxide nanoparticles and copper(II) phosphate trihydrate nanosheets for suppressing the root fungal disease in watermelon caused by *Fusarium oxysporum* f. sp. *niveum*.

##### Other Nanomaterials as Nano-Pesticides

Numerous studies have been conducted on the use of nanomaterials as nano-pesticides, which are different from the above-stated materials [122,123]. Fusarium wilt is considered as a soilborne and seedborne disease commonly seen in chickpea due to the pathogen *Fusarium oxysporum* f. sp. *ciceri*. In [122], chitosan and chitosan nanocomposites were examined as antifungal agents against the pathogen *Fusarium oxysporum* f. sp. *ciceri*, in vivo and in vitro. Of the examined materials, chitosan, chitosan-zinc oxides nanocomposites, and chitosan copper oxide nanocomposites were observed to be very efficient against the pathogen at different concentrations (50.0, 100.0, and 200 μg/mL). Both chitosan-based nanoparticles and chitosan-silver-based nanocomposites have been noted to be reasonably efficient, however not effective as conventional pesticides. All nano-sized materials confirmed better antifungal efficiency, prevented the growth of pathogen, and promoted the chickpea plant growth relative to untreated plants.

### 4.2. Negative Impact of NMs in the Soil Environment

**Figure 6:**
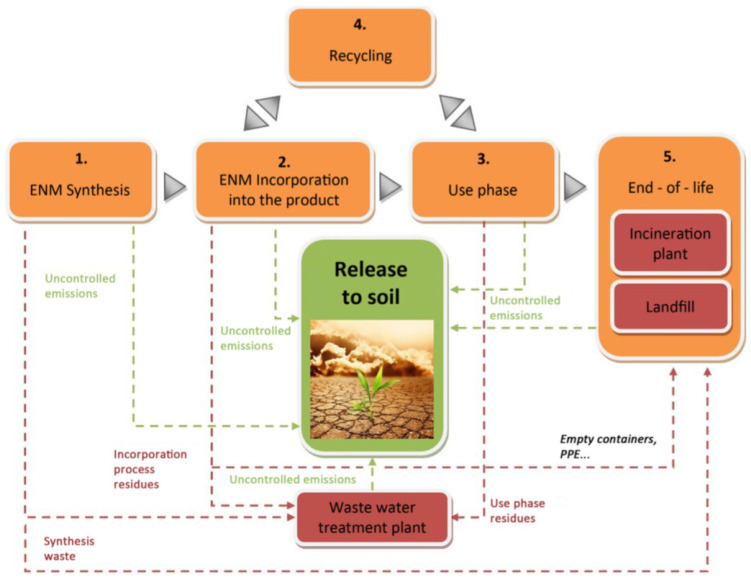
Overall life cycle path examination. Green arrows specify the discharges to the soil and red arrows designate the engineered nanomaterial (ENM) discharge paths ending up in end-of-life treatments or waste management, from which discharge to soil could also happen. Reproduced with permission from [124]. Elsevier, 2018.

**Figure 7:**
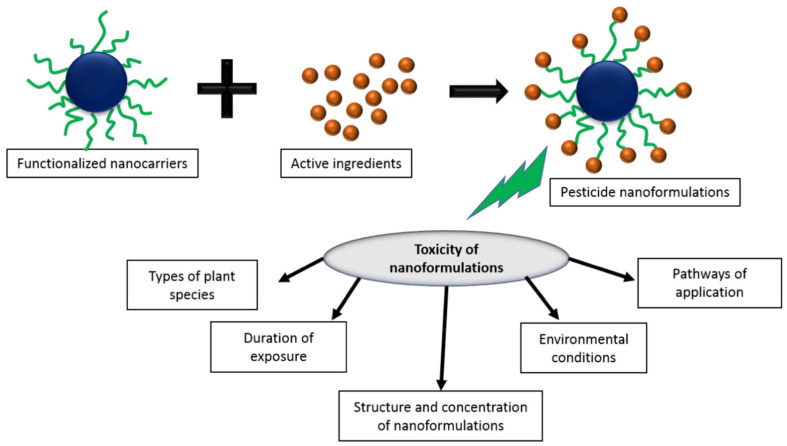
Various factors influencing the pesticide nano-formulation toxicity. Reproduced with permission from [112]. Elsevier, 2018.

During the course of the life cycle of a nano-enabled product, various situations can cause the discharge of engineered nanomaterials, which may in turn affect the ecosystems in a way hard to foresee. Recognizing the paths by which engineered nanomaterials can enter the various ecological compartments is the initial step in the impact evaluation procedure. Figure 6 demonstrates a map of various processes that could possibly result in discharges during the life cycle of a nano-enabled product, concentrating on those reaching the soil compartment [124]. The engineered nanomaterial fabrication and inclusion into the product are generally controlled processes carried out in dedicated facilities, lessening the hazards on discharges. During the utilization and end-of-life phases, certain nano-enabled products might contribute a genuine danger for soil. Figure 7 presents the various factors influencing the pesticide nano-formulation toxicity. The broad discharge of nanomaterials into the surroundings and food chain might pose a human health risk as well. Some studies confirmed that engineered nanomaterials could reduce root elongation, seed germination, impair cell division, damage root cell membranes, and harm plant biomass [125,126,127]. The commercial-scale use of chemical-based fertilizer may lead to permanent damage to plants, soil microbial flora, mineral cycles, and soil structure, as well as negatively influence food chains across the ecosystem, resulting in genetic mutations in forthcoming consumer generations. Different studies have been performed on nanomaterial interactions with edible plants and their probable implications in the food chain [128].

Over the past 10 years, more studies are investigating the toxicity and bioaccumulation of nanomaterials in soil. On the other hand, studies related to this topic are rare, relative to the studies examining the aqueous ecotoxicology of nanomaterials. Earlier studies in this field concentrated on as-prepared nanomaterials and included the bioaccumulation of silver and copper oxide nanomaterials in earthworms [129] and lower bioaccumulation of cerium oxide nanomaterials in corn [130], as well as titanium oxide and zinc oxide nanomaterials in wheat [131].Various studies have recently stated the toxicity of iron oxide nanomaterials on clover [132], accumulation of cerium oxide nanomaterials in soybean roots [133], and the adjustment in localization of nutrients in corn susceptible to cerium oxide nanomaterials [134]. The stronger interaction of most nanomaterials with soils with respect to heteroaggregation with soil dissolved organic matter and minerals increases doubts regarding the ecological significance of ecotoxicological levels of nanomaterials in any non-soil-based medium [135].

We examine the adverse impact of nanomaterials such as carbon nanotubes, silver nanomaterials, copper nanomaterials, titanium dioxide, and iron oxide nanoparticles in the soil environment in the following section.

Ecotoxicological tests for determining the harmfulness of nanoparticles to soil organisms could be performed using various exposure media (e.g., water, food, and soil) (Table 3). Furthermore, evaluation of the harmfulness of the corresponding non-nanosized material or metal salts is important for determining the extent of toxicity produced by ions generated by nanoparticle dissolution or openly associated to the particle nano-sizes.

#### 4.2.1. Carbon-Based Nanomaterials

Carbon-based nanomaterials such as carbon nanotubes and fullerenes are frequently used in consumer products. However, the carbon nanomaterial mass that reaches wastewater treatment plants is comparatively less, and, different from silver nanomaterials, various evidence suggests that carbon-based nanomaterials are only possibly toxic at comparatively higher contents [142,143,144,145,146,147]. Even though a few studies have confirmed the toxicity at higher concentrations in hydroponics [148] and in vitro, the less amount of ecotoxicological work that was performed in soil medium stated less harmfulness to the terrestrial ecosystem [149]. Until date, advanced carbon-based nanomaterials such as graphene showed comparatively lower harmfulness [149].

Currently, carbon nanotubes are progressively more seen in terrestrial and aquatic environments and possible dangerous effects of these nanoparticles on wildlife and humans are receiving increased research and public attention. It was found that the carbon nanotube concentration in contaminated soil can be almost twenty-fold greater than in water [150,151]. This result points out the significance of ecotoxicological dangers of CNTs in the soil environments.

Earthworms are conventionally considered as suitable indicators of land use effect and the fertility of the soil. The authors of [150] studied the integrated response of a suite of biomarkers from molecular to whole organism endpoints for the evaluation of MWCNTs impacts on *Eisenia fetida* (earthworm) exposed to spiked soil. The test results confirm that the biochemical and cellular responses, e.g., immune cells morphometric variations, lysosomal membrane destabilization, metallothionein tissue concentration variations, and acetylcholinesterase inhibition, indicated high sensitivity to CNT exposure. In [152], fullerenes (C60) and two MWCNTs were added to soil to evaluate their influence on the bioaccumulation and oxidative response of 3,6-dimethylphenanthrene, 3-methylphenanthrene, and phenanthrene, by the *Metaphire guillelmi* geophagous earthworm in tri-, bi- and single-contaminant systems. They noted that the oxidative stress occurred in all three systems, but only high-level carbon nanomaterials in tri-contaminant system triggered substantial harm with extensive malondialdehyde developing in earthworms.

Carbon nanomaterials released to the surroundings could have an indirect impact on the microbes present in the soil. Researchers have demonstrated that the existence of carbon nanotubes in the soil influences microbial carbon, decreasing its content and disturbing the microbes present in the soil [153]. Several studies analyzed the impact of multi-walled carbon nanotube on soil microbes [154]. The test results demonstrate that increased concentration of CNTs restrained the microorganism activity in the soil. Furthermore, carbon nanomaterials also have a definite influence on the biodiversity of microorganisms, which might hinder the development of some fungi and bacteria [154]. The single-walled carbon nanotube concentration was adversely associated with the biomass of soil microbial community, and CNTs in the soil showed a definite influence on the composition of microbial communities [155]. Several studies analyzed the impact of carbon NMs on the microbes present in the soil. Oyelami et al. [156] examined the impacts of an enhancement in the concentration of SWCNT, MWCNT, or fullerene on overall microbial activity over an incubation period of 21 days. The results attained from this research illustrate that the inclusion of the aforementioned carbon NMs had no intense influence on the overall microbial activity, and the overall impact of carbon nanomaterials on soil microbial activity did not demonstrate a particular pattern in the short-term. As carbon-based nanomaterials are generated in huge amounts and are also obstinate to degradation, it is recommended that environmental discharge of carbon nanomaterials be maintained at a lower level until additional information on their long-term ecological fate is acquired.

#### 4.2.2. Silver Nanomaterial

Numerous studies were conducted to analyze the toxicity on silver nanomaterials on soil organisms and plants [157,158,159,160]. Recent studies suggested that the environmental concentration of silver nanomaterial is 0.1 µg/kg/year in sludge treated soil and 1.2–5.1 µg/kg/year in natural soil [161]. Due to the developing concerns about the silver nanomaterial discharges into the surroundings, current ecotoxicology research has concentrated on the soil toxicity of silver nanomaterials. The authors of [162,163] incorporated silver nanomaterials of distinct sizes (silver nanoplates, silver nanowires, and silver nanoparticles) into natural soil and examined their influence on the development and reproduction of the free-living *Caenorhabditis elegans* nematode. Silver nanoparticles and nanoplates were noted to hinder the development and reproduction of *Caenorhabditis elegans*, whereas silver nanowires demonstrated a negligible effect. Of the three nanomaterials analyzed, the silver nanoparticles proved to be most toxic. The study confirmed that the silver nanomaterial shape played a substantial role in the toxicity level. This research also provided a scientific reference for evaluating shape-dependent soil nano-toxicity.

The authors of [164] inspected the behavior of silver NPs in plant–soil systems through 72 weeks of extended soil testing, response of earthworm, and plant metabolic analysis. The earthworms exposed to silver nanoparticles did not demonstrate reproductive failure; however, high oxidative stress along with the diminished synthesis of protein resulted in considerable weight loss. The 72 weeks of extended soil incubation analysis proved that the soil quality decline was prominently contingent on the time and levels of silver nanoparticle exposure. The team stated that the retardation in the availability of nutrients was higher with 50 and 25 mg/kg concentrations relative to the 10 mg/kg level. The reproductive failure and mortality in earthworms were not obvious until 50 mg/kg silver nanoparticle exposure. On the other hand, the earthworms experienced serious oxidative stress because of greater levels of silver nanoparticle exposure (50 and 25 mg/kg), as demonstrated in elevation of stress enzyme activity and lipid peroxidation. In addition, serious harm to chloragogenous tissue layer was noticed in earthworms subjected to silver nanoparticles. Thus, the aforementioned study reinforced the foundation for forthcoming researchers to perform focused research works with soils that are constantly subjected to contamination by silver nanoparticles over an extended period of time.

Physicochemical properties of nanoparticles affect their environmental fate and toxicity, and studies examining this are important for a general approach towards a complete and satisfactory environmental risk valuation. The authors of [165] examined the effect of surface coating (charge) and size of Ag nanoparticles on the toxicity (survival, development, and production of cocoon) to the *Lumbricus rubellus* earthworm. The silver nanoparticles coated with bovine serum albumin and related biological molecules demonstrated greater uptake from the soil, resulting in increased potential for toxicity in organisms.

#### 4.2.3. Copper-Based Nanomaterial

Research on metallic engineered nanomaterials has demonstrated that nanoparticulate forms of metal are generally less harmful to soil organisms than their dissolved counterparts [166]. Earthworms exposed to metal-based engineered nanomaterials demonstrate comparable modes of toxicity to those known for dissolved metals, including oxidative stress and increased metallothionein [167].

**Figure 8:**
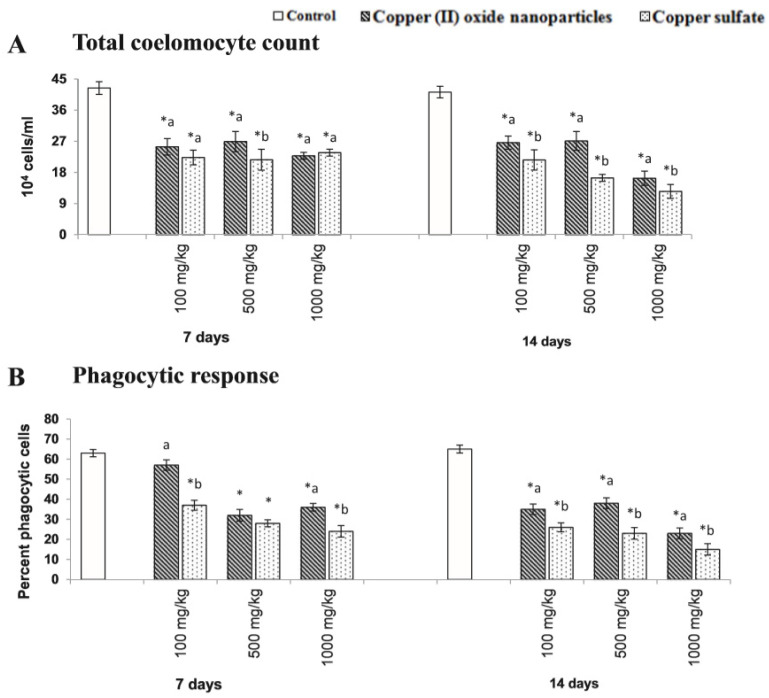
Total coelomocyte count (**A**) and phagocytic response (**B**) of coelomocytes of *Metaphire posthuma* exposed to 100.0, 500.0, and 1000.0 mg of copper nanoparticles and copper sulfate per kg soil for seven and fourteen days. Asterisk (*) signifies the data considerably different from control (*p* < 0.05). Different letters (a and b) signify the substantial difference between the copper nanoparticle and copper sulfate treatment sets for each experimental concentration. Reproduced with permission from [168]. Elsevier, 2018.

Tatsi et al. [167] studied the toxic influence of copper oxide engineered nanomaterials with distinct chemical coatings on *Eisenia fetida* in fresh soil, and subsequently after one year in aged soil. In the two experiments, *Eisenia fetida* was exposed for fourteen days to copper oxide nanomaterials at minimal concentrations of 1000 and 200 mg Cu/kg dry weight and compared to copper sulfate. Thus, they demonstrated certain coating-dependent variations in engineered nanomaterial toxicity to *Eisenia fetida*, which also changed after one year of soil ageing. Gautam et al. [168] demonstrated that the copper sulfate and copper oxide nanoparticles severely influenced the characteristic immunological status of *Metaphire posthuma*. The treatment of copper oxide NP and copper sulfate led to a reduction in total coelomocyte density and phagocytic response of the coelomocyte of *Metaphire posthuma*, as shown in Figure 8.

Mwaanga et al. [140] proved that the abilities of copper, copper oxide, and zinc oxide nanoparticles to cause oxidative stress is dissimilar in various soil media. Therefore, the oxidative stress levels caused by copper, copper oxide, and zinc oxide nanoparticles in artificial and urban soils are diverse even at similar concentrations. They also demonstrated that the soil organic matter had an enormous effect on the impacts of the nanoparticles in producing oxidative stress in earthworms. In addition, they confirmed that there is a relationship between nanoparticle concentration and metal ion accumulation in soils, and this accumulation was considerably greater for nanoparticles than bulk materials. Therefore, the results suggest that it is the form in which the nanoparticles exist which can lead to serious oxidative stress.

#### 4.2.4. Titanium Dioxide Nanomaterial

Severe concerns have been expressed about possible dangers of synthesized titanium dioxide nanoparticles [169,170,171,172,173,174,175,176], despite their benefits [177,178,179,180]. Small-sized titanium dioxide nanoparticles (7 nm) have been observed to be extremely harmful to *C. elegans* as compared to large-sized particles (45 nm) [181]. As compared to non-nano-sized particles, titanium dioxide nanoparticles were observed to be more harmful to earthworms [182] and nematodes [138]. However, both non-nano- and nano-titanium dioxide remarkably diminished the reproduction success and growth of *C. elegans* [138]. The authors observed that the duration of exposure is very important in the determination of whether the nanoparticles are harmful, and the feeding parameters were enhanced by the existence of lower doses of titanium dioxide nanoparticles in a hormetic-like way.

**Figure 9:**
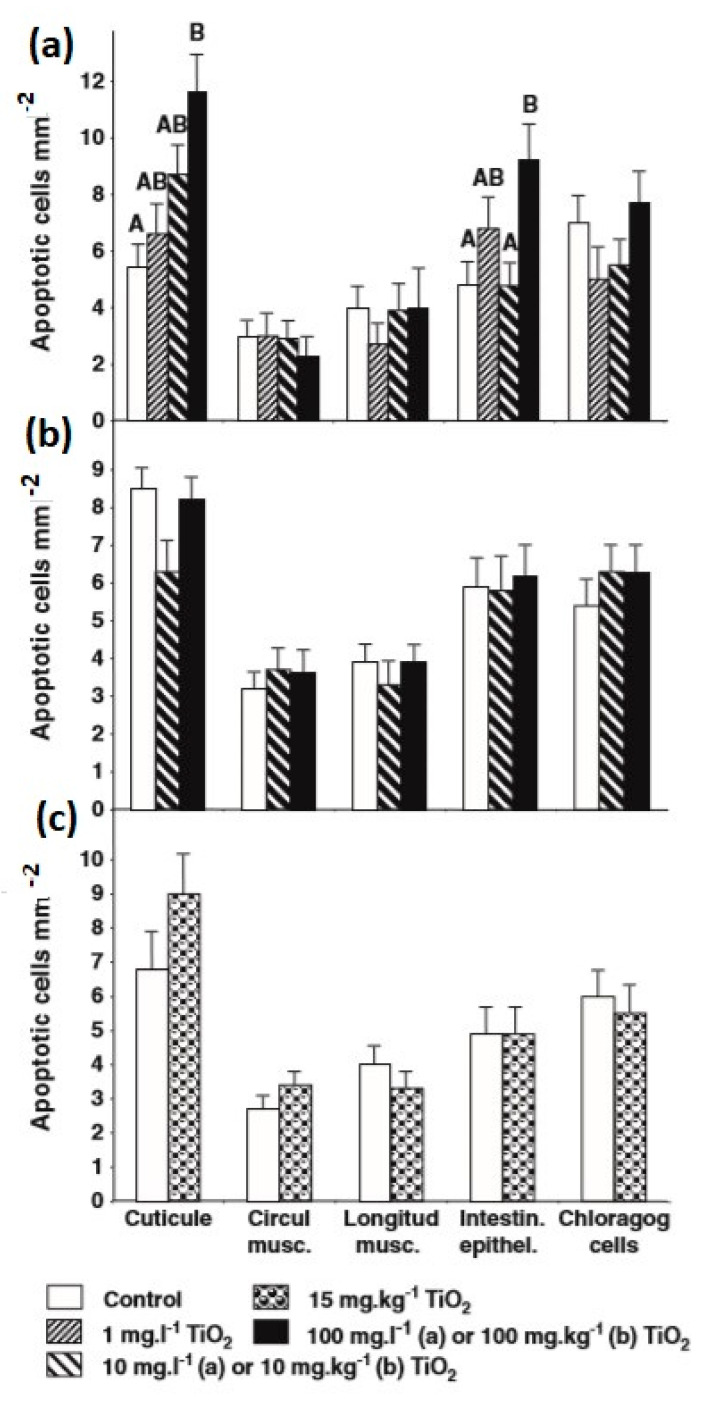
Apostain-positive cell mean densities of various tissues of *Lumbricus terrestris* earthworm exposed to: (**a**) 0.0, 1.0, 10.0. and 100.0 mg/L of titanium dioxide nanocomposites in water for seven days; (**b**) 0.0, 10.0. and 100.0 mg/kg of titanium dioxide nanocomposites in a single food batch eight weeks prior to examination; and (**c**) 0.0 and 15.0 mg/kg of titanium dioxide nanocomposites in soil for four weeks. Error bars signify the standard error of the mean. Within each tissue type, bars having same letters are not substantially different. Reproduced with permission from [184]. Elsevier, 2011.

In the case of *E. fetida*, titanium dioxide nanoparticles led to reproductive harmfulness at 1000 mg/kg dry natural soil, while titanium dioxide microparticles did not influence the reproduction [182]. Furthermore, titanium dioxide was noted to be the most harmful metal oxide nanoparticle for earthworms, as compared to ZrO_2_, SiO_2_, and Al_2_O_3_ nanoparticles. The earthworms *E. fetida* and *E. andrei* were exposed to 5-, 10-, and 21-nm titanium dioxide nanoparticles in field soil and artificial soil [141]. No impacts were noted in the two soils on reproduction, growth, and survival, up to 10,000 mg titanium dioxide/kg soil. It was found that the cocoon production of earthworms was reduced in artificial soil and sand with rising concentration of titanium dioxide; however, it was remarkably dissimilar from the control in the artificial soil examination at 1000 mg/kg [141]. Titanium dioxide nanoparticle bioaccumulation was not seen in *Lumbricus terrestris* earthworm when exposed by means of water, soil, and food [183]. Figure 9 presents the Apostain-positive cell mean densities of various tissues of *Lumbricus terrestris* earthworm exposed to different conditions. Even though apoptosis can be seen in the maximum exposed tissues (e.g., intestine and cuticle), the nanoparticles did not pass the tissue barrier, which prohibited the bioaccumulation.

#### 4.2.5. Iron Oxide Nanomaterial

Magnetite nanoparticles are noted to have remarkable applications: arsenic removal from water, cell separation, separation of DNA, etc. [185,186,187,188,189]. Surface functionalized Fe_3_O_4_ nanomaterials are progressively being utilized in several environmental applications, raising concerns on their fate and ecological impacts [190]. Despite of their benefits [191,192], there are several concerns about the possible risks of iron oxide nanomaterials [193,194,195].

Most nanoparticles including magnetite enter the surroundings with no appropriate former treatment, which could lead to different effects on soil organisms [196,197,198,199,200,201,202,203]. Therefore, the authors of [204] examined the effects brought about by magnetite nanoparticles on *Eudrilus eugeniae* earthworm. The earthworms were permitted to interact with various magnetite nanoparticle concentrations and the impact of these nanoparticles was examined by observing the phenotypic changes, followed by histology and inductively coupled plasma optical emission spectrometry (ICP-OES) analyses. The histological examination confirmed the effect of magnetite nanoparticle exposure on the gut disintegration, epithelium erosion, and fibrosis of the circular muscle. ICP-OES analysis confirmed the magnetite nanoparticle accumulation in the earthworm body. Therefore, this research confirmed the adverse effects of magnetite nanoparticles against the *Eudrilus eugeniae* earthworm, suggesting that discharge of nanoparticles to the surroundings must be regulated.

Valerio et al. [205] addressed the impacts of TiO_2_, Fe_2_O_3_, and ZnO nanoparticles in the survival, reproduction, and body mass change of *Eisenia fetida*. The earthworms were subjected to rising concentrations of each nanoparticle in an amended soil, and the total and bioavailable Fe, Ti, and Zn were assessed in an aerobic incubation test of sixty days. They found that the Fe_2_O_3_ nanoparticles remarkably diminished the earthworm survival, whereas Fe_2_O_3_^−^ and TiO_2_ nanoparticles considerably reduced the reproduction relative to the ZnO nanoparticles.

## 5. Regulations

It is certain that state-of-the-art nanotechnology has been effectively used in numerous areas for the well-being of humans [206,207]. However, any novel unverified technology has some disadvantages. There exist some anxieties about the possible dangerous effects of nanomaterials on human health and the environment [208,209,210,211,212]. There are several reasons to believe that the utilization of nanomaterials is developing. Thus far, nanotechnology is serving significantly to enhance and revolutionize several technologies in addition to industrial sectors: biomedical, environment, energy, photovoltaic, sensing, and catalysis [213,214,215,216,217]. On the other hand, because of the absence of appropriate disposal strategies, the level of nanomaterials in the environment is constantly increasing. Adequate strategies and regulations for the utilization, in addition to the disposal, of nanomaterials must be developed to avoid complications associated with nanomaterial use. Ecological authorities, engineers, scientists, and non-governmental and governmental organizations must theorize about the influence of nanomaterials.

Trying to prevent nanoparticles from continuing to migrate elsewhere once the required work is done, or understanding their exceptional toxic features relative to the bulk form, challenges the important guidelines of prevailing laws. Several studies were conducted on the exposure limits of nanomaterials [218,219,220,221,222]. In addition, certain organizations have suggested tentative occupational exposure limits for nanomaterials. As an illustration, the National Institute for Occupational Safety and Health (NIOSH) in the United States proposed recommended exposure limits of CNTs as 1 μg/m^3^ for 8 h time-weighted average [223]. Furthermore, manufacturers must make available thorough information (e.g., processing, manufacturing methods, specific chemical identity, available health and safety information, production volume, use, exposure, and release information) on nanomaterials to the United States Environmental Protection Agency (US EPA) for evaluation to make sure that products never cause any danger and/or ecological hazards. Moreover, the British Standard Institute (BSI) has recommended nanomaterial “standard levels” for insoluble, extremely soluble, and substances categorized as hazardous as 0.066, 0.50, and 0.10 × the occupational exposure limits of the corresponding micro-sized material, respectively. In addition, the suggested BSI standard level for fibrous nanomaterials is 0.01 fibers/mL. The standard level for metal oxides, metals, and bio-persistent granular nanomaterials (density above 6000 kg/m^3^) is 20,000 particles/cm³ as per the German Institute for Occupational Safety and Health (IFA), and, in the case of bio-persistent granular nanomaterials (density below 6000 kg/m^3^), it is 40,000 particles/cm³. For provisional fibers, the suggested standard level by IFA is 10,000 fibers/cm³. On the other hand, such occupational exposure limits of nanomaterials might not be satisfactory for defense from health hazards. Consequently, it is desirable to retain the exposure limit as low as feasible by performing appropriate control measures such as signage, labeling, and safety datasheets. As stated by the European Union regulation 1272/2008, nanomaterials regarded as dangerous must be categorized and labeled appropriately. Through the Cefic Long-Range Research Initiative project, Read et al. charted the governance landscape for nanotechnology by taking into account the present supervisory frameworks, standardizations, schemes of reporting, and best practice regulation [224]. Additionally, the voluntary structures in European nations for submission of data on nanomaterials is serving to collect data concerning toxicity levels and awareness in manufacture and market distribution [225]. Consequently, it is extremely important to carry out appropriate life-cycle assessment besides the risk assessment evaluations for nanomaterials prior to extensive utilization [226,227,228].

## 6. Future Prospects

The rising number and advancement of improved nanomaterials, along with the excessive development of commercial nano-technological products demands an evaluation of the potential risks and impacts of their discharge into the soil system. As soon as the nanomaterials reach the soil ecosystem, the physicochemical characteristics of nanomaterials could be extremely varied with time.

Proper assessment of environmental nanosafety is urgently needed; however, there still exist gaps in obtaining complete knowledge on the probable interactions between soil and nanomaterials for properly establishing the impacts created over the entire life cycle of each type of nanomaterial. The forecast of adverse effects and environmental risks caused by the environmental transformation of nanomaterials should be established for determining the variations in their reactivity and surface properties. The main technique for understanding the transformation process of nanomaterials in soils is the examination of the nano-bio-ecosystem interactions that happen at interfaces between the biological and environmental systems and the surfaces of nanomaterials. The aforementioned interactions are powerfully affected by the nanomaterial intrinsic properties and the conditions and properties of the environment.

Toxicity is considered to be directly reliant on the exposure route. The fate and mobility of nanomaterial transformation products through the soil environment might contribute a possible danger to numerous ecosystems. Soil, as a unique natural resource for the plant and animal community maintenance, including human life, needs appropriate management and conservation for ensuring its proper functioning. Anthropogenic activities normally aggravate the quality of soil and are accountable for severe deterioration of the ecosystem, which subsequently leads to the decrease in soil productivity and biodiversity. Accordingly, knowledge and information about the properties and nature of each soil and the climate of the precise location must be considered for evaluating the possible toxicological implications developed from the interactions along with the transformations of these nano-sized materials in the soil system. The evaluation of the ecotoxicological impact of the nanomaterials would assist the development of regulatory statements depending on suitable and precise methodologies. As a consequence, the information attained from these analyses could be used for implementing “safety-by-design” methods for producing nanomaterials that could lessen the adverse effects of nanomaterials on the soil system.

## 7. Conclusions

We analyze the recent developments in the role of nanomaterials as: (1) nano-pesticides (for improving the plant resistance against the biotic stress); and (2) nano-fertilizers (for promoting the plant growth by providing vital nutrients). We mostly examine the impacts of nanomaterials such as iron oxide NPs, nano-zerovalent iron, titanium dioxide (TiO_2_), cerium oxide, nanohydroxyapatite, carbon nanotubes (CNTs), silver nanomaterials, and copper-based nanomaterials. It is shown that certain metals such as Zn are crucial, and therefore test organisms must have a minimum concentration of metal in their body, which might be controlled to a consistent limit subsequent to enhanced absorption. Simultaneously, the concentration of metals must not exceed the toxic levels. With the constant advancement in technology, nanomaterials can be extensively utilized for environmental remediation, and the synergism with plants could additionally enhance the remediation efficiency of soils polluted with metalloids and metals. Nanomaterials can change plant carotene and chlorophyll, enhance the plant photosynthesis rate, improve the protein levels of plant, improve fertility of soil, increase growth, and diminish the availability of heavy metals and toxicity to plants. Certain nanomaterials are combined with plants for remediating soils polluted with metalloids and metals. On the other hand, at high concentrations, these nanomaterials might be harmful to plants, primarily leading to diverse leaf toxicity reactions, inhibition of growth, etc. Some nanomaterials at higher concentrations can also cause harmful effects to soil organisms such as earthworms. These nanomaterials can affect the earthworm growth, survival, and reproduction.

In general, this advanced technology of using the nanomaterials as nano-fertilizers and nano-pesticides is considered as promising, and it might rapidly find its way into commercial applications. To accomplish this, additional studies are required on its toxicity to soil and plants. The adverse impacts of nanomaterials in plants may include biological membrane damage, variation in sub-cellular metabolism, slow growth, reduced rate of photosynthesis, plant growth hormone decrease, chromosomal abnormalities, etc. Thus, it can be observed that nanomaterials can enhance as well as disturb the soil ecosystem. It can influence the environmental remediation system by averting the development of secondary derivatives, causing the decomposition of certain toxic contaminants by zero-waste operations, and eliminating additional soil pollution by changing the contaminants from labile to non-labile phases. Further, this nanotechnology will lay the foundation for adaptable and vibrant systems that involve the leading-edge techniques in monitoring and sensing of different types of toxins and destructive chemicals in various environmental media. Different nanoparticles are reported to be harmful at increased doses; however, at optimum concentration, these materials could be advantageous for plant growth and development. However, more studies in this nanotechnology field are required since mild bulk materials can become reactive and toxic materials at nano-levels.

Generally, our observations are: (i) despite the considerable advancements, serious gaps persist to a large extent because of the inadequacy of modeling and field capabilities, and also as a result of the area complexity; (ii) an important awareness gap is the absence of information on the ecological concentrations and dosimetry in general; and (iii) considerable evidence proved that there exist nano-specific effects on the surroundings with respect to toxicity, bioaccumulation, and fate, but they are not constant across nanomaterials, types, and processes. By diminishing the enormous information gaps on the interactions of nanomaterials, it will be possible to obtain suitable approaches as regards the applications, processing, and regulation of nanomaterials in the upcoming years.

## Figures and Tables

**Table 1:** Positive impacts of nanomaterials on plants.

Sl. No.	Nanomaterial	Effects	Impacting Factor	Properties	References
1	Nano zerovalent iron (nZVI)	nZVI acts as rice seed priming agent for enhancing the plant vigor	nZVI	20 mg/L	(Guha et al., 2018 [70])
2	Nano-titanium dioxide	Nano-titanium dioxide altered the proteins of soybean (*Gycin max (L.) Merrill*), chlorophyll, and carotenoid, as well as enhanced the photosynthesis rate	Nano-titanium dioxide	100–300 mg/kg	(Singh et al., 2018 [71])
3	Nano-titanium dioxide	The nanomaterial increased the seed germination rate and the rate of plant growth	Nano-titanium dioxide	<100 nm	(Singh et al., 2018 [71])
4	Nano hydroxyapatite (NHAP)	The presence of nanomaterial remarkably enhanced the ryegrass biomass. The rate of separation of lead from the soil by ryegrass was also increased	Nano-hydroxyapatite	5 g/kg	(Jin et al., 2016 [72])
5	Cerium oxide nanoparticles	The nanoparticle significantly enhanced the height of the plant, biomass, and chlorophyll content	Cerium oxide nanoparticles	500 mg kg^−1^	(Rico et al., 2015 [73])
6	Nanoscale zerovalent iron	The low nanomaterial concentration energized the development and root growth of the plants.	Nanoscale zerovalent iron	10–320 μmol/L)	(Li et al., 2015 [74])

**Table 2:** Different studies based on nano-pesticide application.

Sl. No.	Nanomaterials	Concentration	Pathogen	Plant	Application Type	Suppression of Disease	Ref
1	Silver	100 mg/L	*Colletotrichum* sp.	Pepper	Foliar and pretreated	Yes	(Lamsal et al., 2011 [113])
2	Silver	100 mg/L	*R. solani*	Rice	Foliar	Yes	(Nejad et al., 2016 [105])
3	Copper oxide	500 to 1000 mg/L	*Fusarium oxysporum* f. sp. *niveum*	Watermelon	Foliar	Yes	(Elmer et al., 2018 [114])
4	Single walled carbon nanotubes	500 mg/L	*Fusarium poae* and *Fusarium graminearum*	Wheat plant	Foliar	Yes	(Wang et al., 2014 [115])
5	Multi walled carbon nanotubes	500 mg/L	*Fusarium poae* and *Fusarium graminearum*	Wheat plant	Foliar	Yes	(Wang et al., 2014 [115])
6	Reduced graphene oxide	500 mg/L	*Fusarium poae* and *Fusarium graminearum*	Wheat plant	Foliar	Yes	(Wang et al., 2014 [115])

**Table 3:** Toxicity to the soil invertebrates caused by metal nanoparticles.

Sl. No.	Nanoparticles	Tested Species	Concentration Range	Media of Exposure	Duration	Endpoints	Ref
1	Silver	*Caenorhabditis elegans*	0.1–1 mg∙L^−1^	K-media	24 and 72 h	GST activity, Development of ROS, and reproduction	(Lim et al., 2012 [136])
2	Gold	*E. fetida*	5–50 mg gold/kg dry mass	Artificial soil	28 days	Reproduction, growth survival, and gene expression	(Unrine et al., 2010 [137])
3	Titanium dioxide	*Caenorhabditis elegans*	24–239 mg/L	Water	24 h	Growth, survival, feeding, and reproduction.	(Wang et al., 2009 [138])
4	Cerium oxide (CeO_2_) nanoparticles (NPs)	*E. fetida*	41–10,000 mg Ce/kg	Soil	Survival (at Day 28) and reproduction (at Day 56)	Survival, reproduction	(Lahive et al., 2014 [139])
5	Cu, CuO, and ZnO	*E. fetida*	100–500 mg/kg	Urban and artificial soils.	14 days	Superoxide dismutase (SOD) activity, Glutathione (GSH) activity	(Mwaanga et al., 2017 [140])
6	TiO_2_	*E. fetida* and *E. andrei*	20–10,000 mg/kg	Artificial soil and Field soil	48 h (avoidance), 28 days (reproduction), 18 weeks (growth), 14 days (survival)	Avoidance, reproduction, growth, survival	(McShane et al., 2012 [141])
